# Randomised clinical trial of an intensive intervention in the primary care setting of patients with high plasma fibrinogen in the primary prevention of cardiovascular disease

**DOI:** 10.1186/1756-0500-5-126

**Published:** 2012-03-01

**Authors:** Juan José Rodríguez Cristóbal, Carlos Alonso-Villaverde Grote, Pere Travé Mercadé, José Mª Pérez Santos, Esther Peña Sendra, Anna Muñoz Lloret, Cristina Fernández Pérez, Domingo Bleda Fernández

**Affiliations:** 1 Medicina, Area Básica Salud Florida Sud, Parc dels Ocellets, L'Hospitalet del Llobregat, Barcelona, Spain; 2 Institut Català de Ciències Cardiovasculars, Centre Superior d'Investigacions Científiques, Barcelona, Spain; 3 Laboratorio Analisis Clínicos. Centro Asistencia Primaria Just Oliveras, L'Hospitalet del Llobregat, Barcelona, Spain; 4 Departamento médico, Abbott Healthcare, Barcelona, Spain; 5 Unidad de Apoyo a la Investigación, Hospital Clínico San Carlos, Madrid, Spain; 6 Hospital de Viladecans, Viladecans, Barcelona, Spain

**Keywords:** Fibrinogen, Cholesterol, Cardiovascular risk factors, Primary prevention

## Abstract

**Background:**

We have studied the possible effects of an intensive lifestyle change program on plasma fibrinogen levels, in patients with no cardiovascular disease, with elevated levels of fibrinogen, normal cholesterol levels, and a moderate estimated risk of coronary heart disease (CHD) and we have also analysed whether the effect on fibrinogen is independent of the effect on lipids.

**Results:**

This clinical trial was controlled, unblinded and randomized, with parallel groups, done in 13 Basic Health Areas (BHA) in l'Hospitalet de Llobregat (Barcelona) and Barcelona city. The study included 436 patients, aged between 35 and 75 years, with no cardiovascular disease, elevated levels of fibrinogen (> 300 mg/dl), cholesterol < 250 mg/dl, 218 of whom received a more intensive intervention consisting of advice on lifestyle and treatment. The follow-up frequency of the intervention group was every 2 months. The other 218 patients followed their standard care in the BHAs. Fibrinogen, plasma cholesterol and other clinical biochemistry parameters were assessed.

The evaluation of the baseline characteristics of the patients showed that both groups were homogenous. Obesity and hypertension were the most prevalent risk factors. After 24 months of the study, statistically significant changes were seen between the adjusted means of the two groups, for the following parameters: fibrinogen, plasma cholesterol, systolic and diastolic blood pressure and body mass index.

**Conclusion:**

Intensive intervention to achieve lifestyle changes has shown to be effective in reducing some of the estimated CHD factors. However, the effect of intensive intervention on plasma fibrinogen levels did not correlate with the variations in cholesterol.

**Trial Registration:**

ClinicalTrials.gov: NCT01089530

## Background

This manuscript is a translation of our already published manuscript [1]. Fibrinogen can be considered an independent cardiovascular risk factor (CVRF) [2,3]. However, several studies have shown a correlation between cholesterol (CT) and fibrinogen levels [4]. Also it has been argued that the elevated levels of fibrinogen may be influenced by environmental factors, diet, smoking, excess weight and physical exercise [5]. Various clinical and epidemiological studies have described the implications of the elevated plasma fibrinogen values as CVRF in coronary, cerebral disease and peripheral arteries. The Northwick Park Heart study describes a relationship between high values of plasma fibrinogen and the risk of coronary ischemia [6]. In REGICOR study [7], it was described as an average of fibrinogen of 2.92 g/l in males and 3.09 g/l in women, the plasmatic value of fibrinogen being highest in the subset of smoking patients. The study published by Gil et al. [8], described an intervention study done in a primary care setting, in patients with an average age of 72.6 years and with several CVRF; the prevalence of hyperfibrinogenemia was found to be 26.5%. Other studies in younger patients with an average age of 57 and clinical manifestations of cardiovascular disease, have found a prevalence of 60% [9,10]. In different epidemiological studies, such as the Yano et al. [11], an increase in cardiovascular morbidity and mortality has been shown in patients with fibrinogen levels above 300 mg/dl.

Lifestyle interventions make a notable impact on some of the modifiable CVRF; in spite of this, there are not many studies who have analysed the effects of these modifications (smoking cessation, diet and physical exercise) on fibrinogen levels. In addition, these studies have been conducted in settings different from ours, and mostly at short term [5,6]. Because of this, we have designed a study of intervention, in the primary care setting, to assess the effect on the fibrinogen levels in a subset of patients with intensive intervention (in frequency and intensity) on changes in their lifestyle, as compared to a control group, according to the usual intervention practiced in the basic areas of health (BHA). The study has been done in patients with fibrinogen levels > 300 mg/dl, total cholesterol < 250 mg/dl and an estimated moderate or high CHD risk according to Framingham [12], adjusted according to fibrinogen levels [12,13] and had a follow up period of 2 years on each subject.

### Aims

Primary aim To evaluate the effect of an intensive intervention to modify lifestyle (hypo caloric diet, smoking cessation and physical exercise) in the fibrinogen levels in patients without cardiovascular disease with hyperfibrinogenemia (> 300 mg/dl), total cholesterol levels less than 250 mg/dl and an estimated moderate or high CHD risk.

### Secondary aim

(a) To assess the effect of this intensive intervention in some of the modifiable Cardiovascular Risk Factors.

(b) To confirm that this effect is independent of the variations of the total cholesterol levels.

## Methods

We designed a randomized, controlled clinical trial, parallel groups, consisting of 436 patients, divided into two groups: a) an intensive intervention group, both in the frequency and intensity of their changes in lifestyle, b) a control group, receiving the standard therapy. The protocol has been described in a previous publication [14]. The study was approved by Jordi Gol I Gurina EECC.

Inclusion criteria

- Patients of both genders, aged between 30 and 75 years, in which in two consecutive analyses, separated by a minimum interval of 15 days, with fibrinogen levels > 300 mg/dl and plasma total cholesterol < 250 mg/dl.

- Agreement to participate in the study, with written informed consent using procedures reviewed and approved by the EECC review board.

Exclusion criteria

- Any lipid-lowering therapy (dietary or pharmacological intervention).

- Local or generalized infection, either acute or chronic.

- History of cardiovascular disease, according to medical records and/or anamnesis.

- Fibrinogen lowering therapies (ticlopidine, fibrates, pentoxifylline)

- Severe clinical pathology (terminally ill patients, dementia, etc.)

### Sample size calculation

We have assumed that:

1. The prevalence of smoking, overweight, obesity and sedentarism in the study population are 28, 48 and 84% [7], respectively.

2. The effectiveness of common interventions are: [5,13]

- Giving Up Smoking Advice: 38%.

- Dietary recommendations for overweight/obese patients: 20%.

- Increase physical activity recommendations in sedentary patients: 30%.

3. Smoking cessation and regular physical activity (measured by means of one unit of sporting activity) reduces, on average, fibrinogen levels by 0.4 g/l, and a diet of 1,000-1,400 cal/day reduces mean fibrinogen from 3 to 2.8 g/l [5], during a complete follow up of one year.

Taking into account the prevalence of the cardiovascular risk factors described, the effectiveness of these interventions, its impact on fibrinogen levels and assuming an additive effect of all of them, we aim to achieve a mean reduction of fibrinogen of 17. 2 mg/dl, after a complete follow up of one year.

4. Intensive intervention will get an average reduction of fibrinogen levels twice as much as the standard one, i.e. a reduction of 34.4 mg/dl, during a follow up of one year.

5. An alpha risk of 5%, 80% (1-beta) power and that the standard deviation of fibrinogen is 55 mg/dl [5].

6. A percentage of 20% withdrawals.

Considering all these assumptions, the study was planned to include more than 436 patients.

### Randomisation

Patients meeting the inclusion and exclusion criteria listed above were selected consecutively from those visited by the participating investigators.

### Random allocation sequence

A blocked random allocation sequence was centrally generated by an statistician.

Blocks contained six participants, so that three of them will receive the usual intervention and three the intensive intervention. The order of assigned interventions within each block was randomised. When a investigator received the informed consent of a patient, he phoned a member of the research team, who assigned participants to the corresponding intervention.

### Description of the groups

Control group. This subset of patients have received advice about their lifestyle (diet, exercise and smoking cessation) according to the practice guidelines of the 'Institut Català de la Salut'(ICS), following nernational consensus [12,15].

Intervention group. An active follow up of this group of patients was done, consisting of:

- Phone calls to get psychologist support, and letters to record each visit with the physician, additional measures to encourage the maintenance of lifestyle modifications, which will be done every 2 months. In each visit, physical activity questionnaires were done, as well as both pharmacological medical recommendations and lifestyle changes [15-17]. A laboratory analysis was done every 8 months (Table 1).

**Table 1:** Treatment of patients with intensive intervention

	Definition	Objectives	Intervention	Periodicity
Smoking	People who has smoked daily, any amount of cigarettes, over the last month.	Smoking cessation	Smoking history Degree of dependency Motivation to give up smoking Clear and tailored advice A follow-up program for those patients who stop smoking Use of TSN or bupropion	2 months

Physical activity	Among the activities that people do over a 24 h period, occupational related practices, leisure and free time are the most important ones.	Increase the physical activity	Interview about physical activities and classify: active, partially active or sedentary.	2 months
				
			Advice to start, increase or sustain physical activities.	

Obesity, overweight	Body mass index (BMI) BMI > 30 kg/m^2 ^= obesity 25- 30 kg/m^2 ^= overweight	IMC 20-25 kg/m^2^	Gradual weight loss 0.51 kg per week Advice healthy diet once objectives are achieved	2 months

Hypertension	SBP ≥ 140 mmHg and/or DBP ≥ 90 mmHg	PA < 140/90 mmHg Diabetics BP < 130/80 mmHg	Dietary measures or pharmacological treatment, according to guidelines	2 months

Diabetes mellitus	Two basal glycemias in venous serum ≥ 126 mg/dl	HbA_1C _< 7%	Dietary measures or pharmacological treatment according to guidelines	2 months

*HbA_1C _*glycated hemoglobin, *BP *blood pressure; *SBP *BP systolic diastolic; *SBP *diastolic blood pressure; *TSN *replacement therapy with nicotine

### Blinding

Due to the nature of the study, patients and physicians allocated to the intervention group were aware of the allocated arm. However, outcome assessors and data analysts were kept blinded to the allocation.

Definitions and methods of measurement of outcome variables:

Administrative data: complete name, address and telephone number, name of the medical center of primary care, medical history, number of medical record, date of registration, date of birth and sex.

Toxic history: smoking habits (daily consumption of tobacco in number of cigarettes), alcohol consumption (g/day).

Pathological history: Arterial hypertension (HBP), diabetes mellitus (DM), dislipaemia, overweight/obesity, chronic obstructive pulmonary disease.

Physical examination: weight (kg), size (cm), Body Mass Index (BMI) (kg/m2), Systolic Blood Pressure (SBP) and Diastolic Blood Pressure (DBP).

Physical activity: classification of the patient, according to the intensity of their physical activity: active, partially active or inactive.

Analytical data: Fibrinogen, Total cholesterol, High density lipoprotein cholesterol (HDL-C) Low density lipoprotein cholesterol (LDL-C), triglycerides, glucose, uric acid, Hematocrit (%), white blood cells and platelets.

Collection data and analysis: design of a data collection form, on which is listed data identification, variables of the study and the frequency of visits.

All patients provided written informed consent.

### Statistical analysis

The initial characteristics of both groups have been compared by means of bivariate techniques: χ 2 test, in the case of proportions, and t Student test, in case of means. Analysis of Covariance was used to calculate the effect of the kind of intervention the final fibrinogen adjusted to the total cholesterol values.

To analyze the possible association between the type of intervention (dichotomous qualitative variable) and response variable (plasmatic fibrinogen levels, quantitative variable), Covariance analysis (ANCOVA) will be used. This statistical technique allows us to obtain 2 regression lines, parallel, that correlates the results of fibrinogen levels post intervention with the individual baseline, in each group of intervention, without a possible influence of the fibrinogen level values pre or post intervention between the 2 groups. This technique is also used to verify that the effect of the intervention in the final fibrinogen levels is independent of the final total cholesterol variations.

## Results

39 of the 624 patients at baseline decided not to participate, 19 were excluded because they did not meet inclusion criteria and 130 have an inappropriate laboratory analysis.

**Figure 1:**
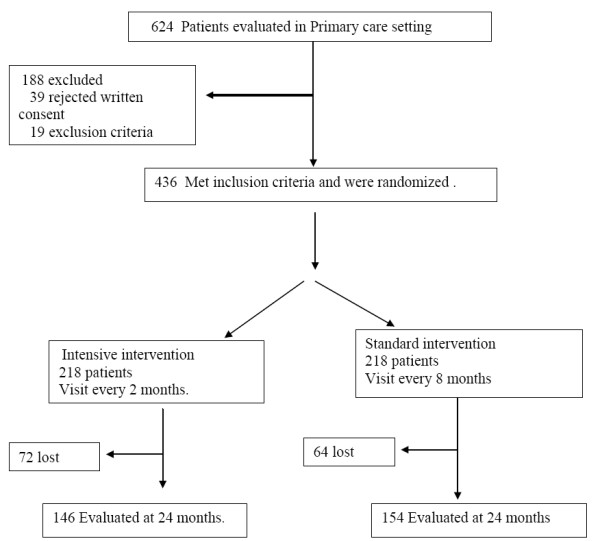
**General outline of the study**.

Of the remaining patients that met inclusion criteria, 438 were randomized and assigned to one of the 2 groups, and followed for a 2 year period. At the end of the 2 years, 72 were lost in the intensive intervention group, 64 in the standard intervention group, (Figure 1). The percentage of patients lost in the follow up was similar for the 2 groups.

Table 2 describes the initial baseline characteristics of the population assigned to each group. Both groups were homogenous, with neither statistically significant nor clinically relevant differences between them. The percentage of individuals who smoked was somewhat higher in the Group of intensive intervention (31.9%) as compared to the standard group (26.8%), while in this last group the number of diabetic patients is slightly larger (15.1% versus 11.9%).

**Table 2:** Baseline characteristics of participants depending on the type intervention assigned*

	Standard intervention (n = 154)	Intensive intervention (n = 146)
Women, n (%)	**98(63.6)**	**92(63.0)**

Men, n(%)	**56(36.4)**	**54(37.0)**

Age in years	58.6 ± 10.6	56.8 ± 10.6

Fibrinogen, mg/dl	368.7 ± 64.5	364.9 ± 59.4

Total cholesterol, mg/dl	210.2 ± 25.5	211.1 ± 26.7

Tryglicerides, mg/dl	116.6 ± 59.6	116.5 ± 54.3

cHDL, mg/dl	55.2 ± 13.1	54.2 ± 12.0

cLDL, mg/dl	134.3 ± 28.6	134.6 ± 26.9

Basal glucose, mg/dl	108.3 ± 33.1	108.5 ± 35.9

**Hematocrit,%**	42.0 ± 3.7	42.1 ± 3.4

Leucocytes, 10e^12^/l	7.6 ± 3.0	7.4 ± 2.0

Platelets, 10e^9^/l	243.0 ± 60.2	252.3 ± 67.6

Weight, kg	76.7 ± 12.9	75.7 ± 13.1

Height, cm	158.7 ± 8.4	158.7 ± 9.8

SBP, mmHg	134.7 ± 18.0	133.8 ± 17.4

DBP, mmHg	81.7 ± 9.4	80.7 ± 9.8

BMI, kg/m^2^	30.5 ± 5.1	30.3 ± 5.8

Obesity, (%)	**(55.5%)**	**(46.8%)**

Tobacco, (%)	**(26.8%)**	**(31.9%)**

Hipertensión arterial(%)	**(41.4%)**	**(42.7%)**

Diabetes mellitus(%)	**(15.1%)**	**(11.9%)**

HbA1c% in diabetic patients	6.9 ± (1.7)	7.1 ± (1.5)

*The values represent mean ± standard deviation, except those in bold, representing number of patients (percentage)

*cHDL *high density lipoprotein cholesterol,

*cLDL *low density lipoprotein cholesterol;

*SBP *systolic blood pressure;

*DBP *diastolic blood pressure;

*BMI *body mass index;

*HbA1C *glycated haemoglobin

Regarding the presence of cardiovascular risk factors, the prevalence of obesity and hypertension were, respectively, 55.5 and 41.4% in the standard group, and 46.8 and 42.7% in the intensive intervention group.

Table 3 shows the results of the analysis per protocol of the main outcomes of the study, according to the type of intervention, after a 2 year follow-up. When analyzing fibrinogen levels, we could find a difference 31.0 mg/dl between the mean adjusted fibrinogen levels, showing a statistically significant (p < 0.001) difference in favour of the intensive intervention. We could also see statistically significant differences in favour of intensive intervention between the adjusted final total cholesterol, SBP, DBP and BMI.

**Table 3:** Results of the per protocol analysis after 2 years of follow-up.

Outcome measure	Intensive intervention (n = 154) mean ± SD	Standard intervention (n = 146) mean ± SD	Adjusted difference mean*	95% CI	p value
Fibrinogen^§^	306.3 ± 57.9	337.6 ± 68.8	31.0	17.0-45.0	0.0001

Total cholesterol	204.4 ± 30.5	224.4 ± 32.0	19.2	12.7-25.6	0.0001

cHDL	61.7 ± 15.1	60.3 ± 14.6	2.1	-0.9 to 51	0.171

cLDL	131.1 ± 28.0	129.6 ± 31.4	-19	-9.9 to 6.0	0.633

Triglycerides	115.1 ± 56.5	119.2 ± 55.4	5.6	-7.4 to 18.6	0.394

SBP	129.6 ± 15.1	136.9 ± 14.8	6.8	2.8-10.7	0.0001

DBP	75.5 ± 9.7	80.4 ± 8.7	4.4	2.0-6.8	0.0001

BMI	29.6 ± 4.8	31.8 ± 4.9	1.7	1.1-2.2	0.0001

HbA1C (diabetic patients)	7.2 ± 1.7	7.7 ± 1.3	0.5	-0.5 to 1.6	0.340

depending on the type of intervention. All comparisons of outcome scores between interventions groups are presented as the difference in means after adjusting for baseline outcome measures

* Difference between groups using covariance analysis after adjusting for baseline outcome measure. Positive values favour intensive intervention

^§ ^is an independent variable

*SD *standard deviation; *CI *confidence interval; *cHDL *high density cholesterol lipoprotein; *cLDL *low density lipoprotein cholesterol; *SBP *systolic blood pressure;

*DBP *diastolic blood pressure; *BMI *body mass index; *HbA1C *glycated haemoglobin

Table 4 shows the result of the 'per protocol evaluation' of 'tobacco consumption' variable according to the type of intervention. The difference in the proportion of smokers at the end the study is statistically significant as compared to the initial for each type of intervention, although we didn't find any significant differences between the proportions of smokers in groups at the end of the study.

**Table 4:** Results of the per protocol analysis after two years of follow-up according to type of intervention for the variable 'tobacco consumption'

Proportion of smokers	Intensive intervention	Standard intervention	Difference of proportions	p value^a^
Before	31.9%	26.8%	5.1%	0.372

After	19.4%	22.1%	2.7%	0.669

Before-after difference	12.5%	4.7%		

95% CI	7. 0-18. 2	1.0-8.4		

p value^b^	0.016	0.0001		

*CI *confidence interval

a Difference between groups, calculated by means of Χ^2 ^(comparison of proportions in independent groups)

b Difference between groups calculated using McNemar test (comparison of proportions with paired data)

**Figure 2:**
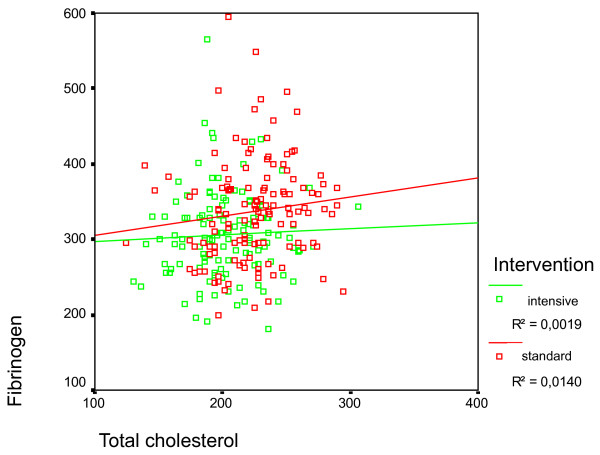
**Relationship between Fibrinogen and final cholesterol study according to the type of intervention**.

Figure 2 shows the relationship between the fibrinogen levels and total cholesterol at the end of the study, according to type of intervention. The Covariance analysis done has not shown any statistically significant relationship between the fibrinogen levels and the total cholesterol at the end of the study.

## Discussion

Baseline demographics did not differ significantly between the 2 groups (Table 2).

The prevalence of some of the cardiovascular risk factors, such as hypertension, diabetes mellitus and smoking are similar to other population studies carried out in our environment [15,18]. However, obesity, in our study, has shown a higher prevalence, possibly due to the characteristics of the population included: patients who visited their physician, mean average age of 57 years, and an estimated moderate or high cardiovascular risk, different from the usual care setting. These results confirms what other investigators have found in relation to obesity [19,20], that it is becoming a major health problem, and desirable implement innovative strategies to prevent and treat it are urgently need.

The lifestyle modification- intensive intervention group, at the end of a 2 years of follow-up period, showed a significant reduction of the weight and BMI, as compared to the standard intervention group.

The results of our patients confirm data from other studies [20,21], showing that lifestyle changes are effective in patients with overweight and obesity.

After 24 months of follow-up, we found a reduction statistically significant of total cholesterol, fibrinogen levels, SBP, DBP and BMI in the intensive intervention group, and showing no differences in other analyzed variables.

These results are similar to the ones found in a study done in 20 centers in a primary care setting in United Kingdom [21], which assessed the effectiveness of the motivational interview to modify the intake of fat, physical activity, SBP and DBP, as well as tobacco consumption in 883 patients with risk high cardiovascular disease. It was obtained a benefit in the intervention group, but these changes could not be neither controlled nor related to the changes achieved in fibrinogen levels.

It is well known that one of the tables used to calculate the estimated cardiovascular risk in primary prevention are tables of Framingham [22,23]. Some authors [13] have proposed to adjust the obtained risk according to fibrinogen levels.

In our study, the fibrinogen levels values in the intensive- intervention group were decreased an average of 31 mg/dl. This would mean that, in the case of a male patient with a 330 mg/dl fibrinogen level, considered an estimated high risk calculated by adjusted Framingham tables by the plasma figures and received intensive intervention, could move to a moderate risk and, therefore, change the therapeutic goal.

## Conclusions

In our study, intensive intervention of lifestyle modifications have been shown effective to reduce some major cardiovascular factors: Fibrinogen, total cholesterol, SBP, DBP and BMI. The effect of the intensive intervention in the fibrinogen plasma values is not correlated with variations the total cholesterol.

Lifestyle modification might play a role in reducing future cardiovascular events in healthy subjects with hyperfibrinogenemia.

## Competing interests

AML acts as a scientific advisor for Abbott Healthcare, SA. All other authors declare that they have no competing interest.

## Authors' contributions

JJRC formulated the research question, designed the study and supervised its conduct together with CAV, PTM, and JMPS. CFP has done the statistical analysis of the EFAP research program. EPS has been working in the patients database. AML and DBF have been involved in the English version of the manuscript. All the authors approved the final manuscript.

## References

[B1] Rodríguez Cristóbal JJ Villaverde Grote CA Flor Serra F Travé Mercadé P Pérez Santos JM Peña Sendra E en representación del grupo EFAP Ensayo clínico de intervención en pacientes con hiperfibrinogenemia en prevención primaria de enfermedad cardiovascular en el ámbito de la atención primaria de salud Clin Invet Arterioscl 2008 20 102 9

[B2] Ernst E Resch KL Fibrinogen as cardiovascular risk factor: a meta-analysis and review of the literature Ann Intern Med 1993 118 956 63 8489110 10.7326/0003-4819-118-12-199306150-00008

[B3] Kannel WB Overview of hemostatic factors involved in atherosclerotic cardiovascular disease Lipid 2005 40 1215 20 10.1007/s11745-005-1488-8 16477805

[B4] Dotevall A Johansson S Wilhelmsen L Associaton between fibrinogen and other risk factor for cardiovascular disease in men and women. Results from the Goteborg MONICA survey 1985 Ann Epidemiol 1994 4 369 74 10.1016/1047-2797(94)90071-X 7981844

[B5] Ernst E Resch KL Therapeutic interventions to lower plasma fibrinogen concentration Eur Heart J 1995 16 Suppl A S47 53 10.1093/eurheartj/16.suppl_a.47 7796831

[B6] Danesh J Collins R Appleby P Peto R Association of fibrinogen, C-reactive protein, albumin, or leukocyte count with coronary heart disease: meta-analyses of prospective studies JAMA 1998 279 1477 82 10.1001/jama.279.18.1477 9600484

[B7] Masiá R Pena A Marrugat J Sala J Vila J Pavesi M Covas M Aubó C Elosua R High prevalence of cardiovascular risk factors in Gerona, Spain, a province with low myocardial infarction incidence. REGICOR Investigators J Epidemiol Community Health 1998 52 707 15 10.1136/jech.52.11.707 10396503 PMC1756647

[B8] Gil B Avilés J Maldonado A Fernández M Factores de riesgo en ancianos. Estudio de 143 cientes An Med Intern (Madrid) 1997 14 495 9 9424138

[B9] Rodríguez JJ Villaverde CA Torelló L Trocho C Tibau N Villaverde AA Relationship between cholesterol and fibrinogen in primary-care areas. 15 the Wonca World Conference 1998 78

[B10] Rodríguez Cristóbal JJ Villaverde Grote CA Tibau Llardén N Juan Babot O Andrades Corrales A Peña Sendra E Relationship between cholesterol and fibrinogen in two populations of different geographical location of Catalonia Rev Clin Esp 2004 204 405 9 10.1157/13064313 15274763

[B11] Yano K Grove J Chen R Rodríguez BL Curb JD Tracy RP Plasma fibrinogen as a predictor of total and cause-specific mortality in elderly Japanese-American men Arterio Throm Vascul Biol 2001 21 1065 70 10.1161/01.ATV.21.6.1065 11397721

[B12] Wood D De Backer G Faergeman O Graham I Mancia G Pyörälä K Prevention of coronary heart disease in clinical practice. Summary of recommendations the second joint task force of European and other societies on coronary prevention Blood Press 1998 5-6 262 9 10.1080/080370598437105 10321437

[B13] Kannel WB Influence of fibrinogen on cardiovascular disease Drugs 1997 54 Suppl 3 S32 40 10.2165/00003495-199700543-00006 9360850

[B14] Rodríguez Cristóbal JJ Benavides Márquez F Villaverde Grote C Peña Sendra E Flor Serra F Travé Mercadé P EFAP Group Randomised clinical trial of an intensive intervention into life-styles of patients with hyperfibrinogenaemia in primary prevention of cardiovascular pathology in primary health care Aten Primaria 2005 35 260 4 10.1157/13072792 15802115 PMC7684374

[B15] Sociedad Española para el Estudio de la Obesidad (SEEDO) Consenso SEEDO'2000 para la evaluación del sobrepeso y la obesidad y el establecimiento de criterios de intervención terapéutica Med Clin (Barc) 2000 115 587 597 11141395

[B16] Maiques A Villar F Brotons C Torcal J Orozco-Beltran D Navarro P Lobos-Bejarano JM Banegas B Ortega S Gil G Solana S Recomendaciones preventivas cardiovasculares en atención primaria. Grupo de expertos del PAPPS Aten Primaria 2007 39 Suppl 3 15 26 19288694

[B17] Woolf S Jonas S Lawrence R Health Promotion and Disease Prevention in Clinical Practice 1996 Baltimore: Williams & Wilkins 177 223

[B18] Medrano MJ Cerrato E Boix R Delgado-Rodríguez M Factores de riesgo cardiovascular en la población española: metaanálisis de estudios transversales Med Clin (Barc) 2005 124 606 12 10.1157/13074389 15871776

[B19] Wadden TA Berkowitz R Womble LG Sarwer DB Phelan S Cato RK Hesson LA Osei SY Kaplan R Stunkard AJ Randomized trial of lifestyle modification and pharmacotherapy for obesity N Engl J Med 2005 353 2111 20 10.1056/NEJMoa050156 16291981

[B20] Shaw K O'Rourke P Del Mar C Kenardy J Intervenciones psicológicas para el sobrepeso o la obesidad (Revisión Cochrane) The Cochrane Library 2005 4 http://www.update-software.com

[B21] Steptoe A Kerry S Rink E Hilton S The impact of behavioral counseling on stage of change in fat intake, physical activity, and cigarette smoking in adults at increased risk of coronary heart disease Am J Public Health 2001 91 265 9 11211636 10.2105/ajph.91.2.265 PMC1446539

[B22] Sans S Fitzgerald AP Royo D Conroy R Gram I Calibración de la tabla SCORE de riesgo cardiovascular para España Rev Esp Cardiol 2007 60 476 85 17535758

[B23] Marrugat J Subirana I Comín E Cabezas C Vila J Elosua R Nam BH Ramos R Sala J Solanas P Cordón F Gené-Badia J D'Agostino RB VERIFICA Investigators Validity of an adaptation of the Framingham cardiovascular risk function: the VERIFICA Study J Epidemiol Community Health 2007 61 40 7 10.1136/jech.2005.038505 17183014 PMC2465597

